# Inequitable Flow of Animals in and Out of Shelters: Comparison of Community-Level Vulnerability for Owner-Surrendered and Subsequently Adopted Animals

**DOI:** 10.3389/fvets.2021.784389

**Published:** 2021-11-11

**Authors:** Lexis H. Ly, Emilia Gordon, Alexandra Protopopova

**Affiliations:** ^1^Animal Welfare Program, Faculty of Land and Food Systems, University of British Columbia, Vancouver, BC, Canada; ^2^The British Columbia Society for the Prevention of Cruelty to Animals, Vancouver, BC, Canada

**Keywords:** adoption barriers, animal sheltering, One Welfare, social determinants, social justice

## Abstract

There is increasing awareness among animal shelter professionals regarding the role of shelters in perpetuating inequities in pet ownership, although the relationship between owner vulnerabilities and animal shelter services is largely understudied. Currently, there is no literature comparing the sociodemographic conditions of communities where surrendered animals originate and communities where they are adopted. The present study compared the “flow” of surrendered animals between originating communities (incoming) and communities where they were adopted (outgoing; *n* = 21,270). To analyze community-level vulnerability, we used the Canadian Index of Multiple Deprivation (CIMD), which has four dimensions of social vulnerability. We found that three of the four CIMD dimensions were significantly different between surrendering and adopting communities (Ethnocultural Composition, Situational Vulnerability (SV), Economic Dependency, but not Residential Instability). For further investigation, we also grouped our analysis by intake groups (small animal *n* = 2,682; puppy *n* = 973; dog *n* = 3,446; kitten *n* = 6,436; cat *n*= 7,733) and found multiple relationships for which the incoming and outgoing CIMD quintiles were different. For example, for both puppies and kittens, the median outgoing SV quintile ranks were statistically significantly lower (less vulnerable) than incoming quintile ranks, with the effect size being moderate (puppy *r* = 0.31, kitten *r* = 0.30; *p* ≤ 0.0025), supporting the concern of the flow of certain animals from more vulnerable to less vulnerable communities. The results of this research provide a basis for understanding potential inequities in the use of shelter services to surrender or adopt an animal. Furthermore, these methods allow animal shelters to assess community needs and create interventions to reduce intake and increase adoption of animals. Finally, these data provide further support that animal sheltering is best considered from a One Welfare perspective.

## Introduction

Animal sheltering organizations are aware of the relationship between human vulnerability and the use of animal shelter services, and express interest in providing more initiatives that target community-level issues (1, 2). Many community-based interventions support owners facing challenges in caring for their animals. For example, animal shelters may offer low-cost or free spay/neuter services for low-income communities (3). Others provide mobile clinics that can reach communities that have difficulty accessing veterinary services (4). Some shelters provide emergency boarding services, wherein owners temporarily board their animals at a shelter or foster home while experiencing a crisis (5). Surrender-prevention programs also support pet owners in areas where they may otherwise relinquish their animals, such as through assistance with paying pet deposits on rental leases, pet food banks, and providing helplines for animal behavior issues (1, 6). Interventions that assist pet owners in pet care ultimately fall under the One Welfare framework, wherein the well-being of humans, animals, and the environment are interconnected (7).

Unfortunately, access to continued pet companionship may not be equal in all groups of people. Pet ownership is more likely in certain demographics such as high-income earners, home owners, and rural residents (8–10). Many studies report that owner-related issues (e.g., financial issues, difficulties finding housing) are more common reasons to surrender pets compared to animal-related reasons [e.g., animal behavior; (11, 12)]. Rose et al. (13) found that neighborhoods in the United States with predominantly African American residents had less availability of pet-inclusive housing, which likely puts people in these communities at a greater risk of surrendering a pet for housing-related reasons. Some recent studies have used measures of social vulnerability, such as the Social Vulnerability Index (SVI) in the United States, which provides a measure of social and environmental inequalities of communities (14). Dyer and Milot (15) compared animal intake and outcomes to social conditions of community members, and found that surrendered pets from more socially vulnerable households were more likely to be euthanized after intake to the shelter. Recently, a striking report by Best Friends Animal Society found that not only did high vulnerability counties within the United States (e.g., low socioeconomic status, racialized population, persons with disabilities) have a higher rate of intake overall compared to nation-wide rates, but also that adoptions, as a proportion of intake, were lower in high social vulnerability areas compared to the national average (16). In our previous work (17), we explored the relationship between community-level vulnerability and owner surrender of animals in British Columbia. This retrospective study used data from the British Columbia Society for the Prevention of Cruelty of Animals (BC SPCA), which consists of 34 animal shelters and 2 foster-based locations. To measure human vulnerability, we used the Canadian Index of Multiple Deprivation (CIMD), which is a measure of social vulnerability similar to the SVI used in previous work in the United States. The results showed that increased vulnerability predicted increased risk of surrender for particular surrender reasons, of particular species or dog breeds, and of particular health statuses upon intake. For example, we found that increased Situational Vulnerability (e.g., higher proportion of low-income individuals, individuals without a high school diploma, single parent families, among other indicators) predicted increased risk of surrendering puppies and kittens compared to cats (the most commonly surrendered animal).

Currently, there is little research investigating the connections between social vulnerability and animal outcomes. In addition to larger societal inequities, one concern within the sheltering field is that animal shelter procedures themselves may be contributing to further inequities (18, 19). Potential barriers include intensive adoption criteria that may encourage discriminatory adoption practices, such as preferentially adopting out to high-income earners who own a home. In a recent questionnaire, 30.5% of shelter organizations reported using pre-adoption home visits to screen adopters (20). The subjectivity of adoption application practices may allow for bias and discrimination against adopters (21), albeit confirmatory research is needed. Similarly, the current system of animal control/animal protection in some countries has disproportionate negative impacts on low-income communities and communities of color, including higher confiscation of animals and lower proportion of animals returned to their owner (22). Currently, animal laws are equivocal and thus may be susceptible to subjectivity, which often leads to over-enforcement for vulnerable communities (22). Perhaps another source of inequity in animal sheltering services comes from the differences between demographics of owners who surrender animals and those who adopt them. Put simply, is it possible that animal shelters are taking from the poor and giving to the rich? This may be occurring in situations where animals are “rescued” from communities where they are free-ranging (i.e., living outdoors) but cared for by community members, and then transported out of their home community for adoption (23). Perhaps it is also occurring locally, as the communities served by animal shelters may vary drastically in social vulnerability.

Despite substantial industry interest in providing more equitable services in animal shelters, research on these topics is lacking. To the best of the authors' knowledge, no study has yet compared vulnerability of communities that use intake services (e.g., owner surrender) compared to those that use outgoing services (e.g., adoption) by connecting the movement of individual animals from intake to adoption. In our previous work, we assessed only the vulnerability of communities surrendering animals. The present study continues this work, with an added layer of assessing community-level vulnerability at adoption. Understanding the “flow” of animals to and from communities of differing vulnerability levels can help animal shelters better understand potential imbalances in the use of these shelter services. Thus, the objective of this study was to understand whether surrendered animals are adopted to communities with the same or different vulnerability levels in British Columbia, Canada.

## Materials and Methods

### Data

This study protocol was reviewed and approved by the University of British Columbia's Research Ethics Board (H20-02704). Permission for data usage was granted by the BC SPCA. The complete dataset can be found in the Supplementary Material. The data used in this study comes from the province of British Columbia, which in 2016 reported a population around 4.6 million (24). The majority of the population identifies as White (64%), with the second most common ethnicity being East and Southeast Asian (18%). Immigrants from China comprise the largest percentage of immigrants to British Columbia (15.5%), followed by India (12.6%), and the United Kingdom [9.6%; (24)]. In 2016, 5.9% of the population identified as Indigenous. In addition, 3.3% of the population are linguistically isolated, meaning they have no knowledge of either of the two official languages of Canada [English and French; (25)]. The median total income of households in 2015 was slightly below $70,000 CAD (24).

This study utilized the CIMD, which is a publicly available measure of social well-being that uses Canadian census data to describe specific dimensions of vulnerability in a small dissemination area [unit of area used by the Canadian census; (26)]. Although the CIMD is a geographically-based index of human social vulnerability, the dataset is also potentially useful as a proxy for individuals living in the dissemination area (27). The CIMD data are available in both raw score and quintiles. This study utilized the CIMD quintile (1–5) score data. Each community is given a raw CIMD score for each factor. Within each factor the scores are then ordered and distributed into five equal quintiles, each quintile holds 20% of the dissemination areas. A higher quintile indicates greater vulnerability based on the indicators for each of the four dimensions. The four dimensions of the CIMD are Ethnocultural Composition (EC), Situational Vulnerability (SV), Economic Dependency (ED), and Residential Instability (RI). The indicators that make up each CIMD dimension can be found in Table 1.

**Table 1:** The four dimensions of multiple deprivation and corresponding indicators for British Columbia (2016).

**Ethnocultural composition**	**Situational vulnerability**	**Economic dependency**	**Residential instability**
Proportion of population who self-identify as a visible minority	Proportion of population that identifies as Aboriginal	Proportion of population participating in the labor force (>15 years)^*^	Proportion of dwellings that are apartment buildings
Proportion of population that is foreign-born	Proportion of population aged 25–64 without a high school diploma	Proportion of population aged 65+	Proportion of persons living alone
Proportion of population with no knowledge of either official language (linguistic isolation)	Proportion of dwellings needing major repairs	Ratio of employment to population^*^	Proportion of dwellings that are owned^*^
Proportion of population who are recent immigrants (arrived in 5 years prior to Census)	Proportion of population that is low-income	Dependency ratio (population 0–14 and 65+ divided by population 15–64)	Proportion of population who moved within the past 5 years
	Proportion of single parent families		

* *Indicates reverse-coded measures. Data are taken from the 2016 Census of Population by Statistics Canada*.

Outgoing animal data were collected from 36 animal shelter locations of the BC SPCA for all animals adopted between January 1, 2016 and December 31, 2019. The BC SPCA is a non-profit organization that operates facilities and programs to improve the lives of animals in B.C. including enforcing provincial animal protection legislation, engaging in advocacy and humane education programs, providing support in the form of pet food banks to the community, and operating 36 animal shelters, 5 animal hospitals/clinics, and a wildlife rehabilitation center (28). In 2020, BC SPCA animal shelters had an intake of ~15,000 animals with an ~90% live release rate [the percentage of animals that exit the shelter alive; (26–28)]. The BC SPCA animal shelters are not the only ones in the province; approximately 17 municipal animal shelters and 110 other rescue organizations also operate in the province (29). The BC SPCA has a largely managed admission system, prioritizing intakes for animal protection and animal control cases and asking surrendering owners to make an appointment [where they may be placed on a waitlist; (30)]. In 2014, the BC SPCA implemented the “Adopters Welcome” program to engage and support adopters and reduce shelter length of stay (31). The open adoptions program aims to reduce barriers to adoption by using conversation-based practices to encourage adoption rather than using traditionally restrictive screening applications (31). The BC SPCA follows this model by asking adopters to fill out an adoption application, which is used as a basis for a conversation of fit of the animal rather than for screening of the adopter (32).

The data collected for this project are similar to those utilized in our previous work (17), although the present study used data prior to 2020 to reduce the possible abnormalities that arose from the SARS-CoV-2 (COVID-19) pandemic. Whereas our previous dataset focused on incoming animals (regardless of their outcome), our present analysis used only data from animals that were surrendered by owners and subsequently adopted from the BC SPCA. Additionally, the current study excluded non-mammalian exotic animals due to low sample size. The outgoing shelter data included information for small animals (including rabbits, rats, guinea pigs, mice, gerbils, hamsters, ferrets, degus, chinchillas, and hedgehogs), cats, kittens (<6 months), dogs, and puppies (<6 months) adopted within this timeframe, and included the location from which the animal was surrendered and the location to which the animal was adopted.

The data were cleaned and analyzed using RStudio version 1.4.1106 (33). The raw dataset included 27,784 observations. To connect animal shelter data with the CIMD, all observations with incoming or outgoing addresses located outside of British Columbia were excluded (*n* = 1,705). Observations were also excluded if either the incoming or outgoing address was non-codable in a geographic information system software Quantum Geographic Information System (QGIS; i.e., incomplete or did not exist; *n* = 4,809). The cleaned, geocoded dataset included 21,270 observations (small animal *n* = 2,682; puppy *n* = 973; dog *n* = 3,446; kitten *n* = 6,436; cat *n* = 7,733).

Although the CIMD used factor analysis to create four independent factors, we verified the independence of the CIMD factors in our dataset with the incoming CIMD scores by using Spearman rank correlations on each possible pair. We found that there were only two relationships that had a weakly positive correlation: RI and EC (*r* = 0.38, *p* < 0.001) and RI and SV (*r* = 0.34, *p* < 0.001).

### Analysis

The change in CIMD quintiles was visualized through histograms and alluvial diagrams, which represent changes in a network structure over time (34). The differences between incoming and outgoing CIMD scores were compared using Wilcoxon Signed Rank Tests. We first performed this test on the entire dataset, and then subsequently performed the test by intake groups for each CIMD factor, resulting in a total of 20 tests. To reduce the possibility of Type I error when performing multiple repeated tests on the same dataset (35), we used a Bonferroni correction, which set the *p*-value for statistical significance at 0.0025. Additionally, a large sample size can lead to small *p*-values resulting from small differences in the data. Therefore, we also chose to evaluate effect sizes through the method of dividing the Wilcoxon Signed Rank test statistic by the square root of the sample size (36). Effect size complements *p*-values to indicate practical significance of the results (37). To determine the size of effect, we followed the guidelines proposed by Cohen (38) where effect of 0.10–0.29 is considered a small effect, 0.30–0.49 is considered a moderate effect, and ≥0.5 is considered a large effect. In order to focus on practical implications, we only discuss results with both statistical significance and at least a small effect size.

## Results

### Ethnocultural Composition

**Figure 1:**
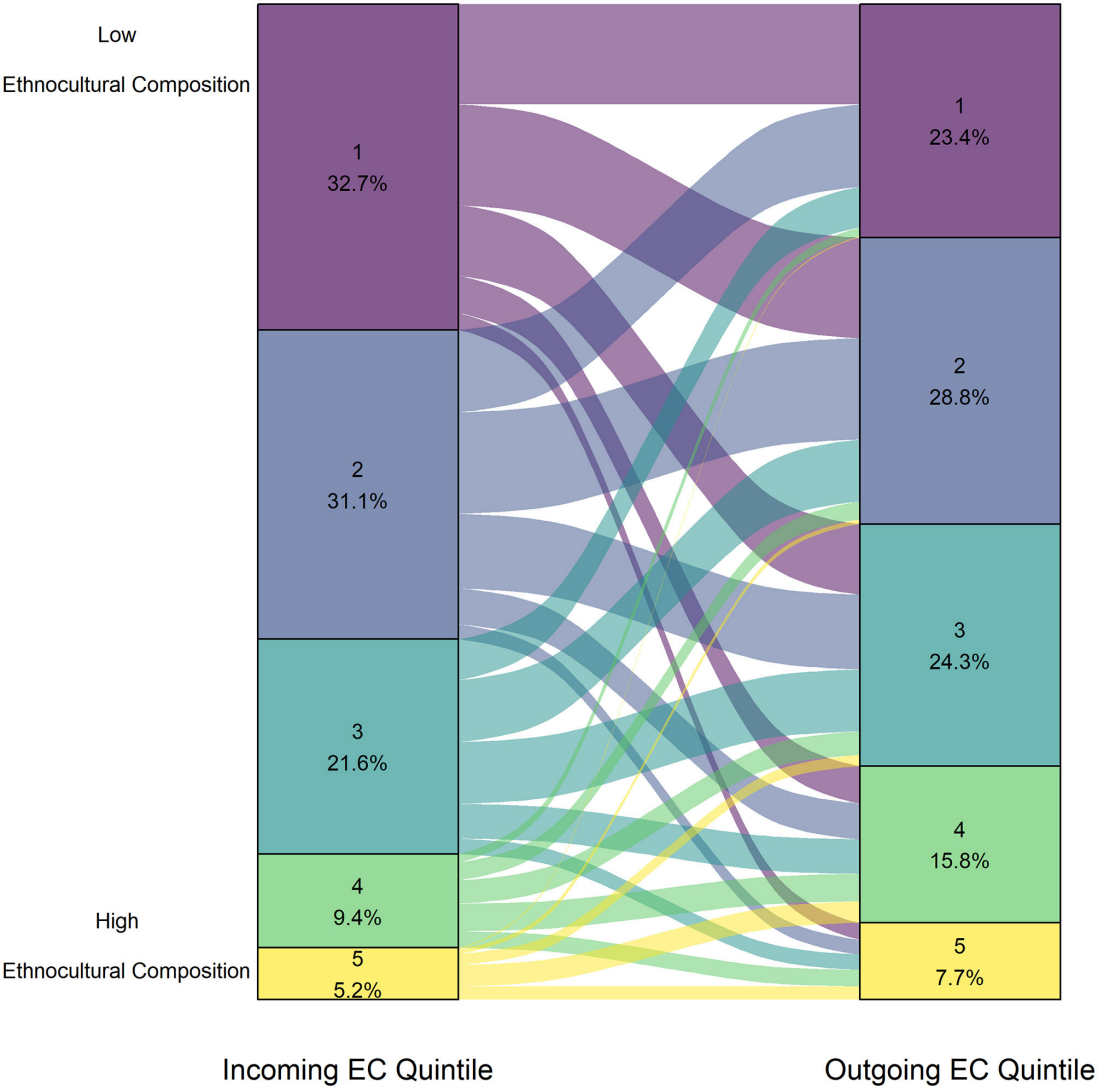
Proportion of animals from each Ethnocultural Composition quintile upon surrender (left axis) and upon adoption (right axis) for all intake groups adopted between January 1, 2016 to December 31, 2019 (*n* = 21,270).

Across all intake groups of animals, the median outgoing EC ranks were higher (more vulnerable) than incoming quintile ranks, with the effect size being small (*p* < 2.20e-16, *r* = 0.14). The distribution of animals incoming and outgoing to each EC quintile is shown in Figure 1.

When comparing by intake groups, the results of the Wilcoxon tests for puppies, kittens, and cats were both statistically significant and had small effect sizes (puppy *r* = 0.23, kitten *r* = 0.21, cat *r* = 0.16; *p* < 2.20e-16; Table 2). For puppies, kittens, and cats, the majority of incoming animals were surrendered from communities with EC quintiles of 1 or 2 (puppy = 77.3%, kitten = 74.5%, cat = 60.6%), while the outgoing proportion of EC quintiles of 1 or 2 were lower (puppy = 59.8%, kitten = 57.0%, cat = 47.3%). The full plots for these three intake groups can be found in the Supplementary File.

**Table 2:** The results of Wilcoxon Signed Rank Test comparing incoming and outgoing Ethnocultural Composition quintiles for each intake group.

**Intake group**	**Wilcoxon D**	***p*-value**	***r* (effect size)**	**Effect size interpretation**
Small Animal	978915	0.020	0.02	No effect
Puppy	60192	<2.20e-16^*^	0.23	Small
Dog	5553455	1.34e-06^*^	0.06	No effect
Kitten	15793665	<2.20e-16^*^	0.21	Small
Cat	24575292	<2.20e-16^*^	0.16	Small

* *p < 0.0025*.

### Situational Vulnerability

**Figure 2:**
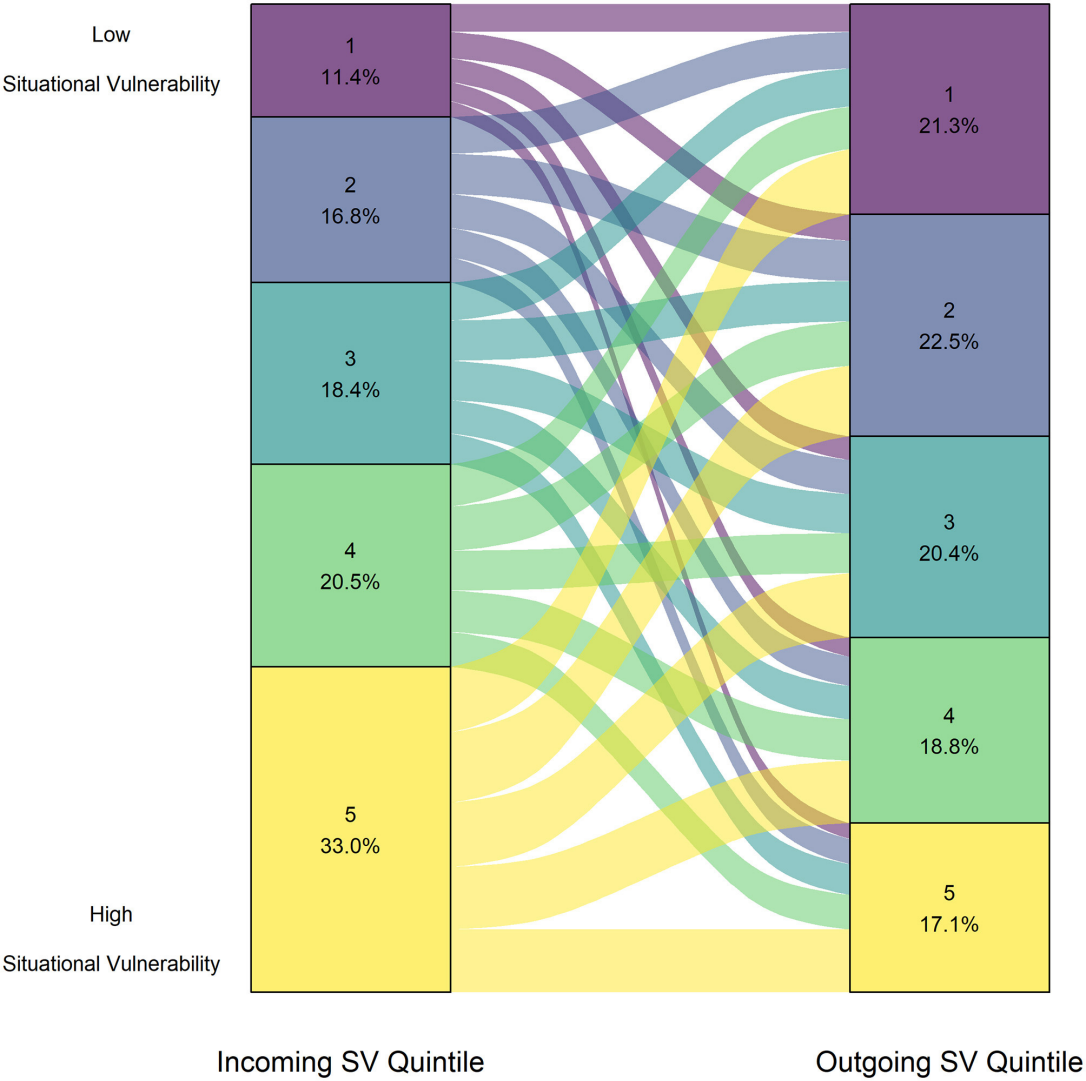
Proportion of animals from each Situational Vulnerability quintile upon surrender (left axis) and upon adoption (right axis) for all intake groups adopted between January 1, 2016 to December 31, 2019 (*n* = 21,270).

The median outgoing SV ranks were higher than incoming quintile ranks across all intake groups of animals, with the effect size being small (*p* < 2.20e-16, *r* = 0.21). The distribution of animals incoming and outgoing to each SV quintile is shown in Figure 2.

**Figure 3:**
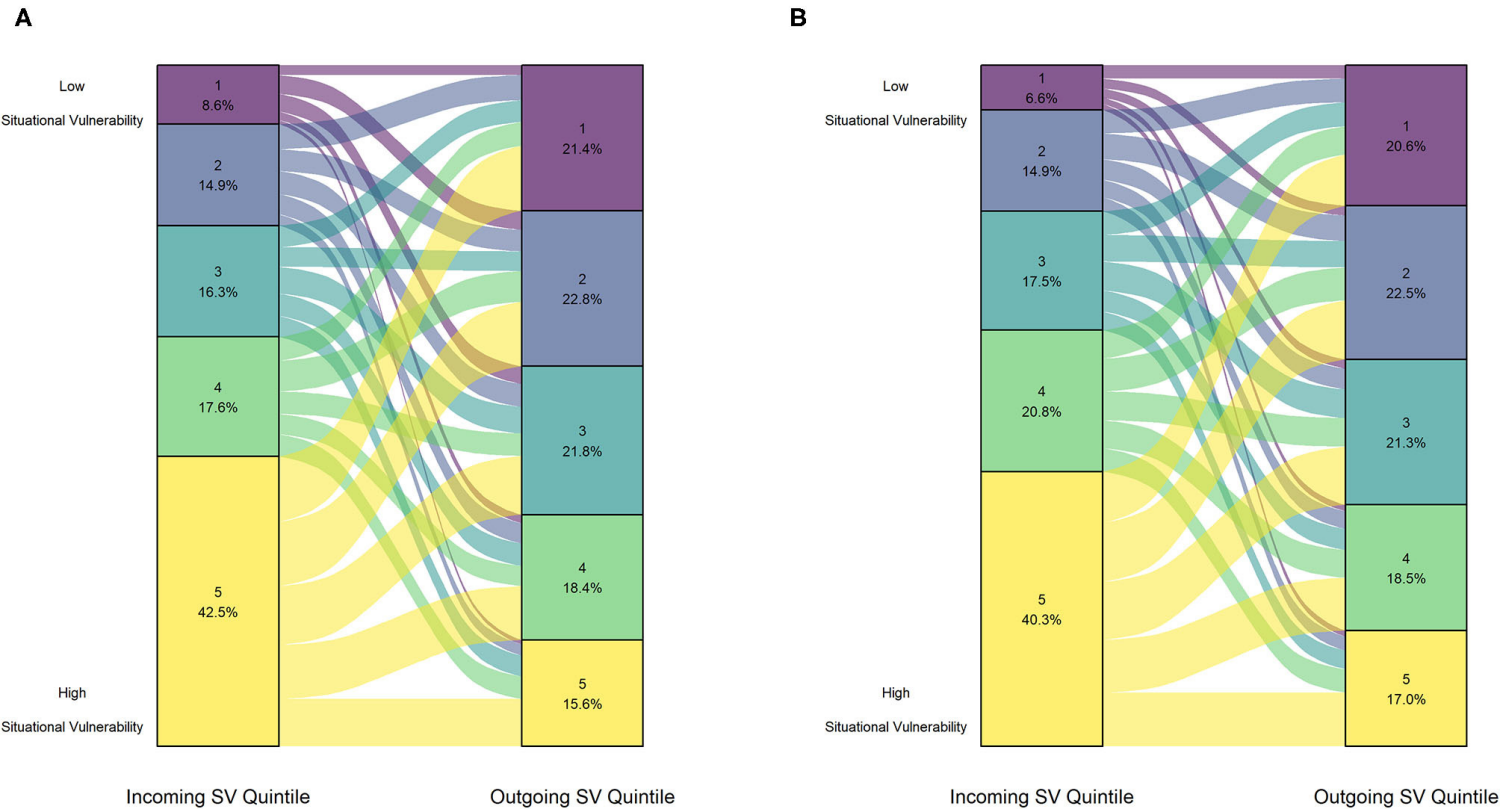
Proportion of animals from each Situational Vulnerability quintile upon surrender (left axis) and upon adoption (right axis) for **(A)** puppies (*n* = 973) and **(B)** kittens (*n* = 6,436).

The results for the Wilcoxon test performed separately by species can be found in Table 3. Notably, for both puppies and kittens, the median outgoing SV quintile ranks were significantly lower (less vulnerable) than incoming quintile ranks, with the effect size being moderate (puppy *r* = 0.31, kitten *r* = 0.30; *p* < 2.20e-16). For both puppies and kittens, the majority of the incoming animals were surrendered from situationally vulnerable communities with SV scores in the 4th or 5th quintile (puppies = 60.1%, kittens = 61.1%), which is shown in Figure 3.

**Table 3:** The results of Wilcoxon Signed Rank Test comparing incoming and outgoing Situational Vulnerability quintiles for each intake group.

**Intake group**	**Wilcoxon D**	***p*-value**	***r* (effect size)**	**Effect size interpretation**
Small Animal	1340980	2.65e-13^*^	0.10	No effect
Puppy	239395	<2.20e-16^*^	0.31	Moderate
Dog	2343185	<2.20e-16^*^	0.16	Small
Kitten	27507833	<2.20e-16^*^	0.30	Moderate
Cat	35976729	<2.20e-16^*^	0.18	Small

* *p < 0.0025*.

**Figure 4:**
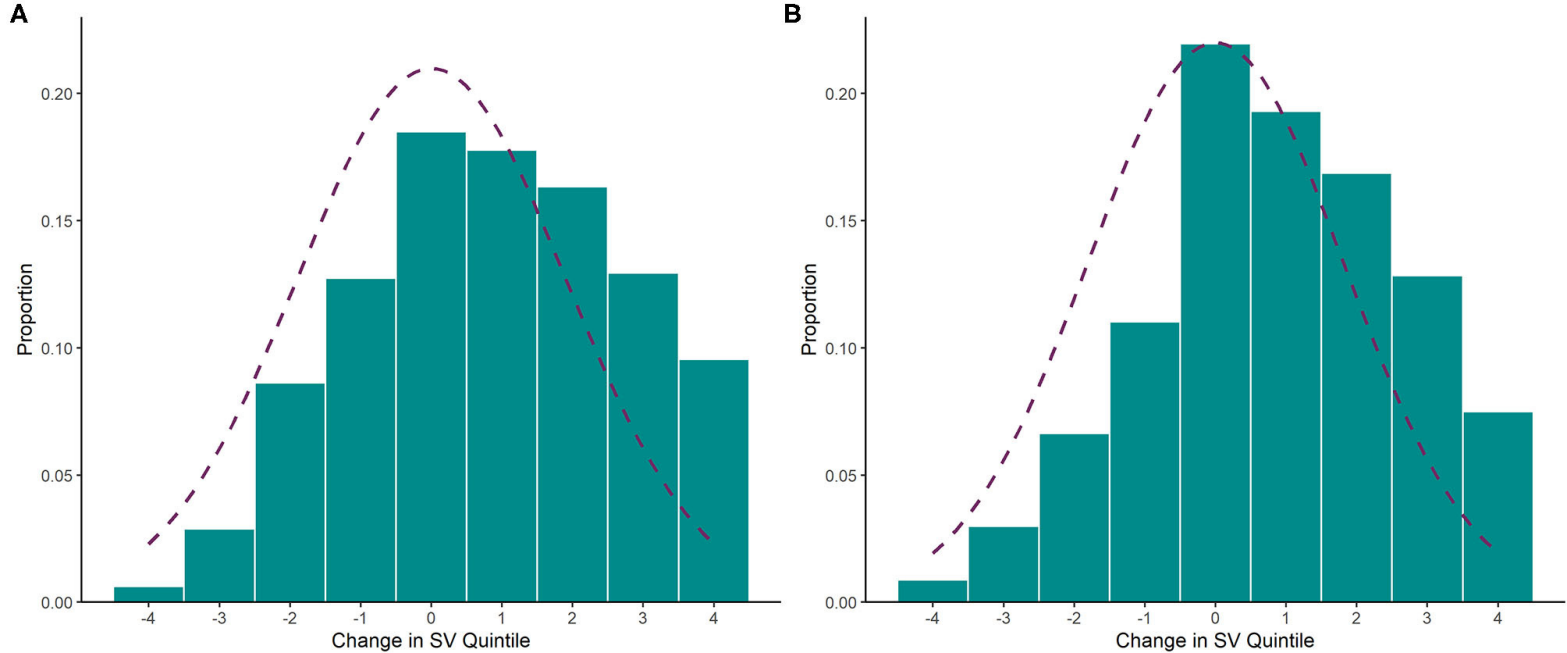
Histogram showing the distribution of change in Situational Vulnerability quintile between the community the originating and adoption community for **(A)** puppies (*n* = 973) and **(B)** kittens (*n* = 6,436). Negative change indicates that the animal moved to a more vulnerable community upon adoption, while positive change indicates that the animal moved to a less vulnerable community upon adoption. The dotted purple line indicates a normal distribution where the mean = 0 and the standard deviation is the same as that of the distribution (1.9).

For further exploration, we calculated the change in CIMD quintile by subtracting the outgoing CIMD quintile from the incoming CIMD quintile. The distribution of change is displayed for puppies and kittens in Figure 4. The Fisher Pearson coefficient showed that both distributions were negatively skewed (puppy = −0.13, kitten = −0.20).

### Economic Dependency

**Figure 5:**
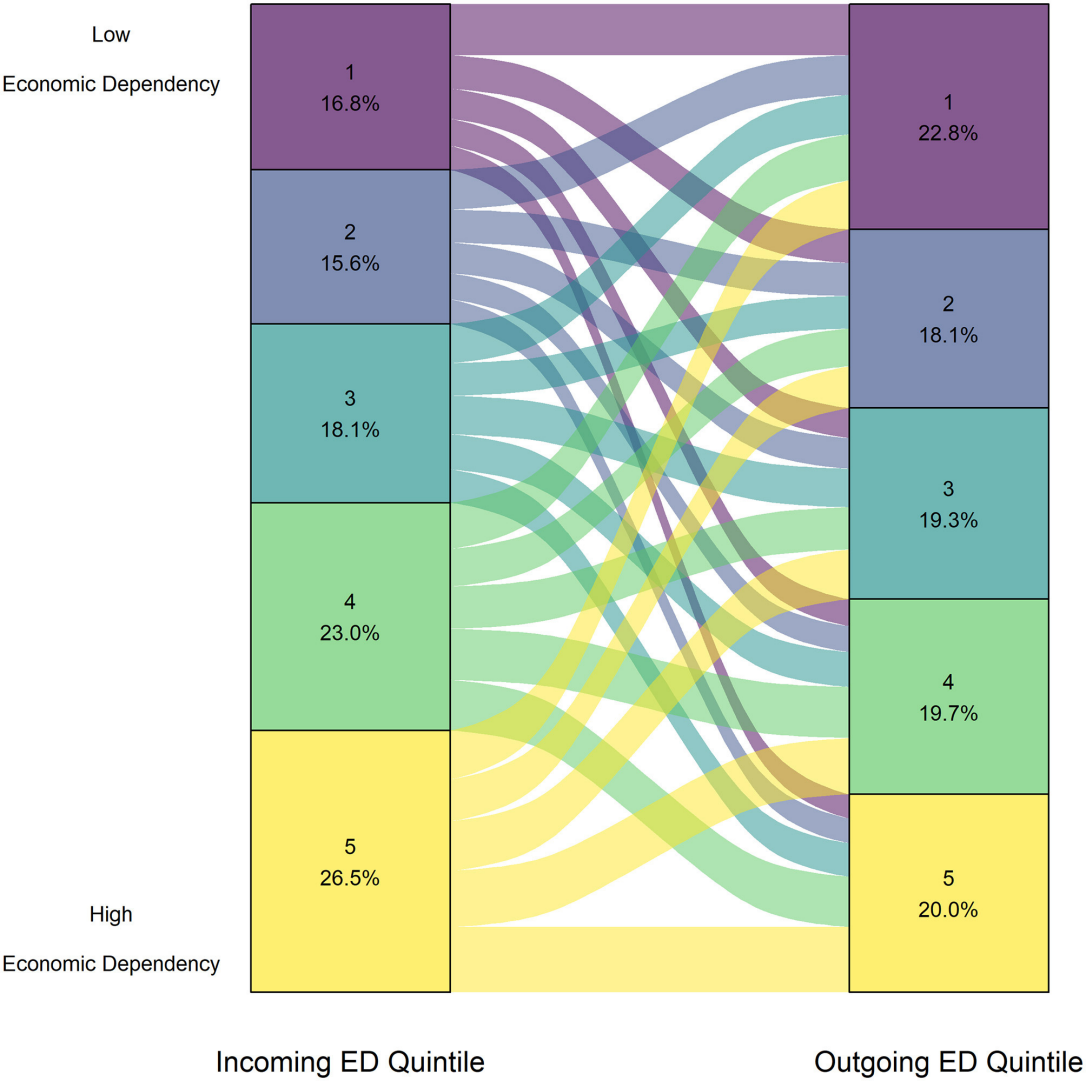
Proportion of animals from each Economic Dependency quintile upon surrender (left axis) and adoption (right axis) for all intake groups adopted between January 1, 2016 to December 31, 2019 (*n* = 21,270).

The results showing the distribution of ED quintiles is shown in Figure 5. Across all intake groups, the median outgoing ED ranks were statistically significantly lower than incoming quintile ranks, with a small effect size (*p* < 2.20e-16, *r* = 0.11).

When the analysis was done separately by intake group, only puppies and kittens showed a statistically significant difference with small effect size (puppy *r* = 0.12, kitten *r* = 0.19; Table 4). For both, the median outgoing ranks were lower than that of the incoming ranks (*p* < 2.20e-16). Both intake groups had the majority of incoming animals in ED quintiles of 4 or 5 (puppy = 52.2%, kitten = 59.5%). Full plots showing the distribution of ED quintiles for puppies and kittens can be found in the Supplementary File.

**Table 4:** The results of Wilcoxon Signed Rank Test comparing incoming and outgoing Economic Dependency quintiles for each intake group.

**Intake group**	**Wilcoxon D**	***p*-value**	***r* (effect size)**	**Effect size interpretation**
Small Animal	1213199	1.13e-5^*^	0.06	No effect
Puppy	182442	9.01e-15^*^	0.17	Small
Dog	1783682	0.027	0.02	No effect
Kitten	24961823	<2.2e-16^*^	0.19	Small
Cat	32748074	<2.2e-16^*^	0.08	No effect

* *p < 0.0025*.

### Residential Instability

**Figure 6:**
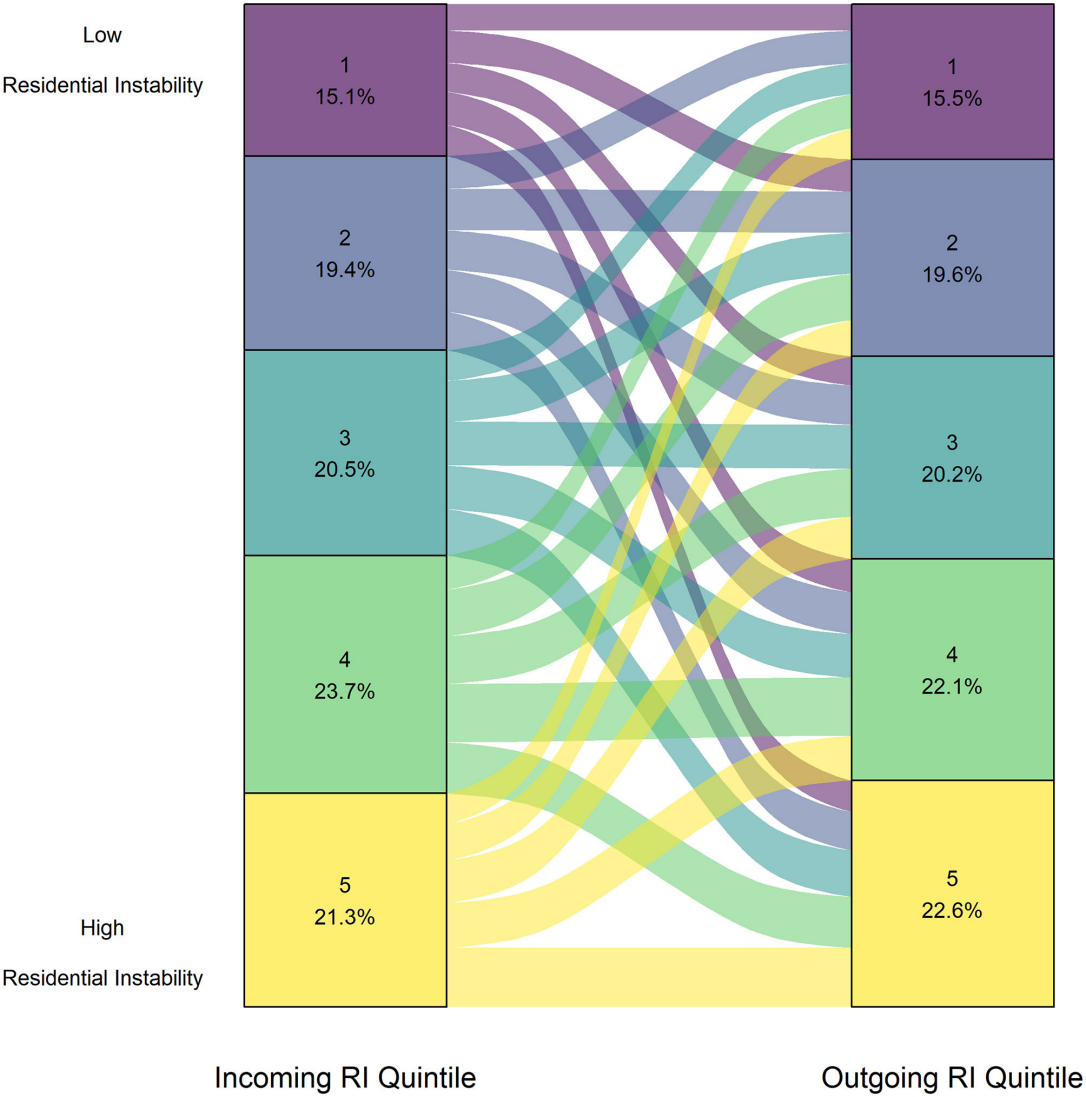
Proportion of animals from each Residential Instability quintile upon surrender (left axis) and upon adoption (right axis) for all intake groups adopted between January 1, 2016 to December 31, 2019 (*n* = 21,270).

For all intake groups of animals, RI was the only CIMD dimension for which the difference between median ranks for incoming and outgoing quintiles were not statistically significantly different and had no effect (*p* = 0.91, *r* = 0.0006; Figure 6).

The results of the Wilcoxon test for RI by intake groups are shown in Table 5. The results of the Wilcoxon test across all intake groups had no effect except for small animals, for which the effect size was small (*p* < 2.20e-16, *r* = 0.14). For small animals, the median outgoing RI quintile ranks were lower than that of the incoming ranks. The majority of incoming small animals were surrendered from communities in the 4th and 5th RI quintile (4 = 20.1%, 5 = 36.1%), whereas the small animals were adopted out to communities in a relatively balanced manner (1 = 16.7%, 2 = 18.5%, 3 = 19.8%, 4 = 22.3%, 5 = 22.7%). The plot showing the distribution of small animals can be found in the Supplementary File.

**Table 5:** The results of Wilcoxon Signed Rank Test comparing incoming and outgoing Residential Instability quintiles for each intake group.

**Species**	**Wilcoxon D**	***p*-value**	***r* (effect size)**	**Effect size interpretation**
Small Animal	1374335	<2.2e-16^*^	0.14	Small
Puppy	471019	0.85	0.004	No effect
Dog	6048219	0.17	0.02	No effect
Kitten	20480374	0.89	0.001	No effect
Cat	7728866	1.88e-11^*^	0.06	No effect

* *p < 0.0025*.

## Discussion

In our previous work, we found that the risk of owner surrender for various reasons, of various species/breeds, and of various health statuses was predicted by the different CIMD factors, showing that community vulnerability affects animal surrender (17). In our present analysis, we tracked the movement of animals from surrendering to adopting communities in order to understand potential imbalances in the use of shelter services. We found that there were multiple statistically significant differences between the incoming and outgoing CIMD quintiles. Our data revealed that in most instances, except for Ethnocultural Composition, the imbalance was largely due to disproportionate intake of animals from more vulnerable communities, rather than an imbalance at adoption. This is the first study to explore the “flow” of animals in this sense and will help animal shelters better understand the use of shelter services by vulnerable communities.

### Ethnocultural Composition

A larger proportion of animals both originated from and were adopted to communities of low EC, indicating less presence of racialized and immigrant populations in both surrendering and adoptive communities overall. However, there was still a large difference between incoming and outgoing EC quintiles; a further disproportionate number of incoming animals were surrendered from areas with low EC, even lower than areas into which animals were subsequently adopted. In Canada, the phrase “visible minority” refers to those who are non-White (39). The phrase “racialized populations” is used to replace the term “visible minority,” in recognition that race is a social construct rather than a biological one (40). Furthermore, in some parts of the country, former “minority” populations now comprise a majority (40). Many immigrants are also from racialized communities, as such, these components constitute the Ethnocultural Composition facet of the CIMD. For our dataset, results may be explained by differences in ethnic composition between rural and urban areas of the province. In Canada, the majority of racialized populations live in large urban areas (24). In British Columbia, the largest urban centers are along the coast of the province, while smaller communities are spread throughout the North and Interior of the province (25). Therefore, in our study, this result may be due to transfer of animals from rural communities to urban communities. The BC SPCA has an active internal animal transfer program that moves ~5,000 animals a year from shelters in areas in the province with overpopulation to shelters in areas with more adoption capacity. The source communities are largely in Northern and Interior British Columbia, where many rural communities have less racial/ethnic diversity. The shelter intake per capita of the Northern region is 12 times higher than that of the coastal metropolitan areas (41). Animals are typically transferred to the coastal metropolitan areas, where the majority of the province's population resides.

As we previously found, puppies and kittens were less likely to be surrendered from areas with high EC; this result is expected as the metropolitan areas of British Columbia are largely comprised of communities with high EC (17). Indeed, previous studies in other regions have found that dog and cat ownership is more prevalent in rural communities (42, 43). Previous research also shows that people in rural communities are less likely to have spayed/neutered their animals (44, 45). Gaps in veterinary services for rural communities may be contributing to increased litters of puppies and kittens that are subsequently surrendered to animal shelters.

Across all animal intake types, the EC quintiles of adopter communities were imbalanced, with a large number of animals being adopted to communities with low EC. One explanation may be that the imbalance stems from differences in the overall likelihood of companion animal ownership between rural and urban communities. In Ontario, Canada, those who live in rural areas or on properties that were greater than one acre were more likely to own pets (9). Owning a companion animal in a rural environment may be easier than in an urban environment due to less restrictive housing policies. While there is limited research directly comparing pet-friendly housing and ethnicity, initial research by Rose et al. (13) found that fewer than half of the landlords in predominantly African American neighborhoods in the United States allowed pets in rental units.

The procedures of the sheltering system itself may pose barriers to adoption. The criteria that are outlined in restrictive pet adoption processes (e.g., owning a home, standalone home, fenced yard, minimum income requirement) are likely to disproportionately affect racialized populations (19), although this area is vastly understudied. Moreover, it is possible that adoption decisions may be impacted by implicit bias, wherein most people have an unconscious bias against individuals of traditionally marginalized groups (46). Implicit bias has been studied in many settings such as health care services (47), law enforcement [including animal control; (21, 48)], and educational institutions (49, 50). As such, it is likely that animal shelters may unconsciously perpetuate societal bias in their intake and adoption procedures.

Questions that remain on less restrictive adoption applications, although not directly discriminatory, may still be subject to differences between cultures. For example, adoption applications may ask questions regarding caretaking behaviors, such as where an animal will spend time and sleep (20). Contemporary pet caretaking behaviors may differ between cultures, as the history of companion animals vary widely (51, 52). One survey in Malaysia found that 87% of respondents reported feeding outdoor-roaming cats, although research in the United States report varied estimates of outdoor cat-feeding behaviors ranging from 10 to 26% of respondents (45, 53, 54). In many national parks, leashing dogs is mandatory, although compliance was found to vary slightly based on country (55–57). One qualitative study found that American residents described several “norms” of pet ownership, such as multiple daily walks and inside access, although these practices do not necessarily reflect pet ownership practices universally (58). For example, crating dogs when left alone is acceptable and encouraged by the Canadian Veterinary Medical Association (59) but is not permitted in many countries like Sweden (60). Thus, the answers to the adoption application may be judged based on the cultural context of the shelter staff member or volunteer, rather than the adopter. Although the BC SPCA implemented the Adopters Welcome procedures prior to data collection, our results show that animals are still disproportionately adopted to communities with low EC. Further research is needed to understand whether conversation-based adoptions are subject to implicit bias or judgements based on cultural context.

Previous research and industry statements have noted that racialized populations are underrepresented in animal welfare professions (61–63). This under-representation may result in animal shelters unknowingly creating uncomfortable environments for racialized populations due to linguistic or cultural differences. Another explanation for our data may be that animal shelters have not placed sufficient effort in reaching out to communities with high EC to offer services, including pet surrender and adoption. The EC dimension also includes measures such as linguistic isolation, wherein one has no knowledge of either of the two official languages of Canada (English or French). Perhaps interventions related to education or pet care resources could be made available in other languages to connect linguistically isolated communities with animal shelter services. In public health literature, language barriers can lead to patients having decreased confidence in the services received (64). Even among Canadians who do speak English as a second language, some report discomfort with seeking health care services, and tend to visit health care services less frequently (65). In the health sector, the term “cultural competency” aims to improve the accessibility and effectiveness of health care for racialized populations, and interventions to improve cultural competency include improving knowledge and attitudes of cultures and increasing diversity of the workplace (66). Comparable literature in the animal welfare field is lacking, although Poss and Everett (67) found that providing bilingual, mobile veterinary services in a county that bordered Mexico and the United States increased use of the services. Future animal welfare research should focus on cultural competency and use of services, particularly for areas with high Ethnocultural diversity.

### Situational Vulnerability

Our study found that puppies and kittens were disproportionately surrendered from communities with high situational vulnerability (e.g., low-income, fewer years of education, high proportion of Indigenous peoples). In our previous study, companion animals from areas of high SV were at an increased risk of being surrendered due to the owner having “too many” animals, surrendering animals that were intact upon intake, and surrendering puppies and kittens (17). Indeed, previous research outlines the relationship between socioeconomic factors and ownership of intact animals. One Australian study found that the greatest intake of puppies and kittens came from unwanted litters (68). Cost is a significant barrier to owners spaying or neutering their animal. White et al. (3) found that pet owners who used low-cost spay/neuter clinic services had significantly lower median income compared to the general population of the United States. Spay/neuter programs are primary examples of animal shelter services aiming to tackle owner-related issues in order to reduce intake to animal shelters (69). In our geographic area of study, there are only two dedicated spay/neuter clinics in the entire province (although there are several organizations operating spay/neuter programs in partnership with community general practices). Disproportionate surrender of puppies and kittens from areas of high SV may be due to the limited presence of spay/neuter clinics and programs. On the other hand, our results revealed that the distribution of SV quintiles for communities that adopted puppies and kittens was relatively equal, suggesting that the BC SPCA's adoption practices posed few financial barriers. In our study, equal SV distribution of adopting communities may indicate that the adoption processes of the shelters did not lead to discrimination based on factors such as income, educational level, and Indigenous status, all of which are factors in determining the SV score of a community. While some animal shelters report collecting “financial means” information from potential adopters as part of the screening process, this concept is dependent on multiple complex factors such as the pet owners' priorities and cost of living. As such, Griffin et al. (20) suggest that measuring “financial means” to screen adopters is not an objective or beneficial measure of the animals' potential quality of life. One example of reducing financial barriers to adoption is low-cost or no-cost animal adoptions. Despite traditional beliefs that low- or no-cost animal adoptions may lead to devaluation of an animal and subsequently lower quality of life for the animal, there is evidence that the adoption fee does not make a difference in subsequent attachment to the pet (70).

### Economic Dependency

The difference between incoming and outgoing ED quintiles was largely driven by puppies and kittens, where most animals were surrendered from high ED communities. The ED factor also indicates that the community has a high proportion of unemployed individuals, which includes those who are collecting a pension, those who are too young to participate in the workforce, and those who are receiving income assistance. As previously mentioned, cost is a significant barrier to accessing veterinary care (4, 71). Although there are limited initiatives that do provide low-income veterinary services in BC, there is still overwhelming need for assistance, particularly in vulnerable demographics such as seniors, who may find it difficult to reach veterinary services (71). While it may be expected that communities with high unemployment are closely related to those with low-income, we did not find that the SV and ED factors of the CIMD were strongly correlated. Job insecurity may lead to working unconventional hours (72), which may lead to challenges raising a puppy or kitten. However, the relationship between employment status and pet ownership challenges has not been widely studied. Overall, high ED indicates potential employment-related challenges that may lead to unwanted litters of puppies and kittens that are subsequently relinquished.

Although the ED dimension indicates non-employment or income from non-employment sources, it also describes the presence of two specific populations—seniors (>65) and children (<15). There are possible challenges that arise from pet ownership for seniors. In senior care homes, pet ownership may be discouraged due to risk of zoonotic disease and extra workload (73). Both dogs and cats may increase risk of falling (74, 75). Older adults with pets could be at increased risk of avoiding or neglecting their own health care due to fear of losing the animal (76). Children and adolescents also experience an increased risk of dog bites (77). The highest risk of dog bites in the United States is reported in children from ages 5–9 (78). However, companion animals also play an important role. Older adults show high levels of attachment to their pets, and they may substitute or complement human companionship following the death of friends and family members (79). Research also shows that pet ownership may buffer stressful situations, improve physical activity, and increase resiliency against depression and cognitive decline (80–83). For children, pet ownership may improve the development of empathy, enhance self-esteem, increase learning abilities and reduce symptoms of loneliness (84–86).

In our study, ED quintiles of adopted animals were relatively equal in distribution, which may indicate that the conversation-based adoption procedures do not discriminate based on family composition or employment status. Questions related to family composition are anecdotally important to animal shelter staff when screening potential adopters. Some animal shelters require a minimum age requirement for children in a home, typically around 4 or 5 years old (20). However, several studies have found that increased number of family members and households containing children are more likely to own a companion animal (9, 42, 87). Families with children, who are interested in owning a companion animal, may seek other means rather than an animal shelter to acquire an animal. Because families with children are likely to own companion animals, perhaps it is more effective for animal shelters to not exclude this demographic from adopting animals, but rather provide resources and support for pet owners.

### Residential Instability

In our dataset, differences between outgoing and incoming RI quintiles had a statistically significant but small effect for small animals only. Housing-related issues are a commonly reported surrender reasons for companion animals (88). A study in Australia found that the most common *owner-related* reason for surrender of adult cats was lack of pet-inclusive housing (89). A scoping review by Coe et al. (88) found that the rental housing and moving issues were the most commonly investigated owner-related reason in primary literature. There is significantly less literature on surrender reasons for species of animals other than dogs and cats. In the United States, Cook and McCobb (90) reported the primary reason for owner surrender of rabbits was the inability to care for rabbits (27%), although housing-related issues were also commonly cited (22%). Ellis et al. (91) found that housing-related issues of the owner were the second most common surrender reason of rabbits in the United Kingdom. A recent report found that only 29% of surveyed property managers allow small animals in their buildings, which potentially increases risk of surrender of these species for housing-related issues (92). Our study suggests that housing issues are a relevant cause for surrender for small companion animal species, which warrants further investigation into the relationship between pet-inclusive housing policies and small animal ownership.

Although the relationship between RI and animal flow was weak for dogs and cats, it is possible that housing-related issues are still barriers to retention of animals in homes for these species on an individual level, as many studies have found that housing-related issues are a significant reason for surrender for dogs and cats (88, 89, 93). In Canada, surveys of multiple animal shelters show that housing is a primary reason for relinquishment (94), with owners citing concerns such as landlord restrictions on pet ownership or high costs of pet-friendly housing (95–97).Whereas the present study did not analyze reasons for relinquishment, our previous work showed that in areas of British Columbia (i.e., Kamloops), RI predicted increased risk of surrender for owner-related reasons—including housing issues—across all species (17). It is possible that housing-related challenges were not captured when comparing RI quintiles between intake to adoption, but may be revealed by alternative (i.e., qualitative) analyses; in fact, previous reports of BC SPCA data have shown that animal owners directly cite lack of pet friendly housing is a significant contributor to cat and dog surrender (98). While the RI dimension captures both neighborhood and familial aspects of housing insecurity, it does not measure pet-specific challenges such as restrictive landlords. Finally, rental housing is becoming a larger proportion of accommodations in British Columbia (99, 100), which may lead to more housing-related challenges for pet owners of all species. As such, housing-related issues are still relevant for other companion animal species and should continue to be addressed by animal shelters.

Animal shelters often survey potential adopters regarding their home environment, and even conduct home visits to personally evaluate the home environment (20). Griffin et al. surveyed 269 animal shelter organizations in the United Kingdom and found that almost half of the adopters' characteristics deemed “most important” by animal shelters were characteristics about the adopter's accommodation, including the type of home, home ownership, the presence of a yard, and other physical characteristics of the home environment. Some of the adopter screening questions described by Griffin et al. (20) were quite specific, such as the shelter asking potential adopters about the type of flooring in their house; however, the only housing-related characteristic that has shown to increase risk of relinquishment is living in an apartment (101). On the other hand, housing type and environment have not been associated with decreased pet welfare or increased risk to human safety (20).

Our study did not find disproportionate outgoing quintiles as a result of adoption across all animals, which potentially suggests that discrimination of adopters is not directly occurring due to housing environments in the BC SPCA sheltering system's adoption processes. This may be due to the implementation of conversation-based adoption procedures at the BC SPCA, where potentially discriminatory housing-related factors such as landlord checks, and home visits were removed. Further research may be necessary to understand the implication of housing requirements on pet adoption, as some continue to use accommodation-related questions to screen potential adopters.

The RI dimension of the CIMD is relevant to the discussion of inequities as pet-inclusive housing is an ongoing topic of concern among both pet owners and animal shelters (88, 92). It may be difficult for shelters to directly intervene in housing-related challenges because this likely requires approaches that change rules and legislation related to pet-inclusive rental agreements or other accommodations (68). Some animal shelters do have initiatives to tackle housing issues. For example, the BC SPCA has educational resources and sample documents for pet owners, property managers, renters, and owners to encourage pet-inclusive housing (98). Other initiatives include paying for pet deposits for renters, and assisting with the construction of fencing so the pet can spend time outdoors safely (102, 103). Some shelters' temporary boarding programs accommodate pets whose owners are in-between housing situations (104); the BC SPCA offers up to 2 weeks of free emergency boarding for such owners. Ongoing research is needed in this area to reduce surrender from communities with high housing insecurity, particularly for homes with small animal species.

### General Discussion and Limitations

Many vulnerable populations are predisposed to risks of multiple vulnerabilities, as such; it is difficult to isolate community vulnerabilities. For example, the most common source of income for those experiencing homelessness in British Columbia is income assistance (99). While housing insecurity is captured by the RI dimension of the CIMD, income assistance is represented by the ED dimension. This relates to public health research that uses the social determinants of health, which are upstream factors such as income, education, employment, housing, and race, that are thought to impact health outcomes (105). The relationship between sociodemographic conditions and health are multifactorial and complex, and do not imply a linear relationship (106). However, much like in public health research, our study provides the basis for further exploration of small portions of the causal web between sociodemographic conditions and animal shelter services.

The use of a geographically based index such as the CIMD may limit the findings as relative to the surrenderers' and adopters' locations rather than the current sociodemographic status of the individual owner. This index may be subject to ecological fallacy, where an inference regarding an individual is based on the findings from a group-level analysis, since an individual living in a dissemination area identified as deprived may not necessarily be vulnerable (107). However, the CIMD uses the smallest unit for which all census data are collected—this same data is not available on an individual level. Thus, we believe that the CIMD is a meaningful proxy as a starting point for understanding challenges that may be faced by individuals in these dissemination areas.

The geographical nature of the CIMD also limits the analysis of the present study to a population-level, as such, these findings may not capture the lived experience of the communities in question. Using population-level analysis is beneficial, as previous studies have used this to identify community needs and create interventions (3); however, further success of community programs could be accomplished by understanding the needs of individuals who may use these programs. Future studies could also use both population-level and individual-level measurements. For example, Spencer et al. (2) used GIS maps to select areas of high intake of stray dogs and used census data and child maltreatment data and subsequently performed qualitative interviews with members of the identified communities to understand possible reasons for high levels of stray dog intake. Understanding the lived experience of individuals in vulnerable communities may increase the efficacy of community-based interventions beyond that of population-level analysis.

This study limited analysis to animals that were surrendered by the owner. For other avenues of intake (e.g., stray animals), analyses were limited because many incoming community animals only have general finder locations (e.g., neighborhood, roads) rather than exact addresses, and therefore could not be geocoded. However, inclusion of other incoming animals may have impacted results. For example, in India, a study found that communities with low socioeconomic status had a higher mean number of free-ranging dogs (57.4) per neighborhood compared to communities with middle (39.8) and high (17.0) socioeconomic statuses (108). Spencer et al. (2) found that communities with high intake (including strays) overlapped with areas with a high-density of child maltreatment cases. Further research could similarly compare proportions of animals through intake and adoption while including other avenues of animal intake.

The data used in this study were only for animals that were both surrendered from and adopted to communities within the province. Transferring animals between shelters (or states in the United States) is a common method to improve animal outcomes, as ownership of pets and adopter preferences vary by region (109, 110). Transfer programs are important for animal shelters with strict euthanasia policies, high intake pressure, and limited capacity for care (111). Transfer of animals may pose challenges to creation of interventions that reduce intake and increase adoption in the source community because of geographic distance between the source and destination communities. Therefore, to effectively serve the immediate community, targeted research in a shelters' served community may be necessary, as communities may differ in demography, legislation, animal shelter services, or culture surrounding pet ownership. Dolan et al. (69) found that reasons for relinquishment in the Los Angeles county differed from that of studies in other communities, possibly due to the mandatory spay/neuter laws in California. Weiss et al. (5) found that the use of animal shelters to re-home pets varied by community. Miller et al. (112) used geospatial techniques to identify communities with high intake to create a targeted intervention that reduced the intake of owned cats. The BC SPCA is a large system of shelters with an established program to transfer within the province, and these findings may differ from that of other types of organizations in different regions. Overall, further research should be conducted in other areas of interest, as understanding an animal shelters' served community could help identify needs and create useful support for pet owners.

Our study may suggest support for the practice of open adoptions as we found only limited evidence of inequity at adoption, albeit experimental data are needed. In 2014, the BC SPCA implemented open adoption practices that may have contributed to the balanced distribution of adopting communities for three of the CIMD factors. The CIMD factor which did show unequal distribution (EC) may be the least impacted by open adoption practices, which are more focused on removing barriers related to factors such as housing, income, and prior pet experiences (31). Open adoption practices do not explicitly address racial, ethnic, or cultural issues such as implicit bias, systemic oppression, or cultural competency. Although these issues are impacted by the removal of other barriers, our study did not directly assess the impact of Adopters Welcome practices on adoption outcomes. Therefore, animal shelters should pursue further direct work regarding services for racialized, immigrant, and linguistically isolated populations. Furthermore, from the present analysis, we do not know the proportion of interested adopters from each quintile who had successful adoptions. It is possible that the proportion of interested adopters at animal shelters varied by CIMD quintile, although the resulting distribution of successful adoptions was even. Future research could explore adoption applications by sociodemographic factors to understand potential avenues of bias in animal shelters and rescues.

In many areas, shelters emphasize adoption of animals in ways typically believed to reduce risk of re-relinquishment (113), which often manifests as restrictive adoption practices. This may be at odds with the principles of “capacity for care,” which broadly include managing intake and outcomes in order to maintain a shelter population that can feasibly be cared for, safeguarding animal welfare and health (114) and decreasing owner and animal stress resulting from relinquishment (112). Capacity for Care (C4C) is also a formal management model that aims to improve the welfare of shelter animals by improving housing and ensuring that populations remain within the capacity of the institution to provide humane care (115, 116). Another primary goal to remain under capacity is to preserve shelter resources to respond to community needs, as animal shelters serve as a safety net for pet owners and animals who need it most (114). To meet the aforementioned goals, animal shelters have also begun providing services that help pet owners and reduce risk of surrender, such as low-cost spay/neuter clinics and pet food banks. Due to the undeniable connection between pet owners and companion animals, actions to safeguard or improve the human-companion animal bond fall under the One Welfare framework, where the well-being of humans, animals, and the environment are interconnected (7). Whether or not animal shelters recognize the One Welfare approach, pet owner-oriented interventions provide evidence that One Welfare strategies do help reduce intake to animal shelters (117). Our study provides further evidence that animal shelters can focus on human support services in order to reduce relinquishment. While further research is needed on adoption services, animal shelters can also consider evaluating their own adoption screening practices to promote non-discriminatory adoption of animals. Overall, animal shelters should continue to explore community-specific methods to support pets, owners, and interested adopters to reach their goal of maintaining a robust safety net and optimizing the mental and physical health of shelter animals.

## Conclusion

Our data showed that, from 2016 to 2019, there were multiple differences in the vulnerabilities between owners' surrendering communities and adopters' communities. However, the imbalance in CIMD quintiles was mainly due to disproportionate surrender of animals from more vulnerable communities, with a notable exception for Ethnocultural Composition. The results add to previous work on social vulnerability and animal shelter services by including comparisons to outgoing communities of animals in order to identify possible barriers or discrimination. Although barriers to adoption were another potential source of inequity, our study locations did not show evidence of unevenly distributed adoption of animals based on most sociodemographic factors. Our findings may be due to open adoption policies enacted specifically to reduce adoption barriers prior to the study period, although this was not experimentally addressed. There was uneven adoption of animals based on Ethnocultural Composition, with a higher proportion of animals being adopted out to low vulnerability communities, which could imply direct or indirect discrimination based on race/ethnicity or culture. However, further research is needed to understand whether the uneven adoption distribution is driven by lack of access to animal shelters, an unwelcoming environment of the shelter, or other factors. As this work is also location-specific, animal shelters and rescues should investigate these differences in their own communities. Identifying CIMD dimensions which are different between incoming and outgoing communities does not necessarily imply a causal association, as the nature of systemic issues of vulnerability is complex. However, the results of this study can be used to help animal shelters reflect on practices related to owner surrender and adoption. Furthermore, the results can help inform interventions to reduce shelter intake and maintain the human-animal bond.

## Data Availability Statement

The original contributions presented in the study are included in the article/Supplementary Material, further inquiries can be directed to the corresponding author/s.

## Ethics Statement

The studies involving human participants were reviewed and approved by University of British Columbia Behavioural Research Ethics Board. Written informed consent for participation was not required for this study in accordance with the national legislation and the institutional requirements.

## Author Contributions

LL, EG, and AP contributed to the conception of the study and the subsequent study design. LL acquired the database, organized and visualized the data, and wrote the first draft of the manuscript. LL and AP performed the statistical analysis. All authors contributed to the manuscript, further revisions, and approved the final version of the manuscript for submission.

## Funding

This research was funded by the Social Sciences and Humanities Research Council (SSHRC) through the Canada Graduate Scholarships—Master's program.

## Conflict of Interest

Data were obtained from the BC SPCA, who is the employer of one of the authors EG. The remaining authors declare that the research was conducted in the absence of any commercial or financial relationships that could be construed as a potential conflict of interest.

## Publisher's Note

All claims expressed in this article are solely those of the authors and do not necessarily represent those of their affiliated organizations, or those of the publisher, the editors and the reviewers. Any product that may be evaluated in this article, or claim that may be made by its manufacturer, is not guaranteed or endorsed by the publisher.

## Supplementary Material

**GitHub for R Code used in the manuscript “Inequitable flow of animals in and out of shelters: comparison of community-level vulnerability for owner-surrendered and subsequently adopted animals”**

https://github.com/lexisly/flow-CIMD

Supplementary Data Sheet 1 Adopted animals from BC SPCA shelters from January 1, 2016 and December 31, 2019.

Supplementary Data Sheet 2 Supplementary alluvial plots showing the flow of animals with small effect size.
