# Supplementary material for: Inequitable Flow of Animals in and Out of Shelters: Comparison of Community-Level Vulnerability for Owner-Surrendered and Subsequently Adopted Animals
Source: Front Vet Sci. 2021 Nov 11;8:784389. doi: 10.3389/fvets.2021.784389 (PMC8635993; doi:10.3389/fvets.2021.784389)

# **Supplementary File – Alluvial Plots**

**Figure 1. Proportion of cats from each Ethnocultural Composition quintile upon surrender (left axis) and upon adoption (right axis) for all intake groups adopted between January 1, 2016 to December 31, 2019 (n = 7,733).**
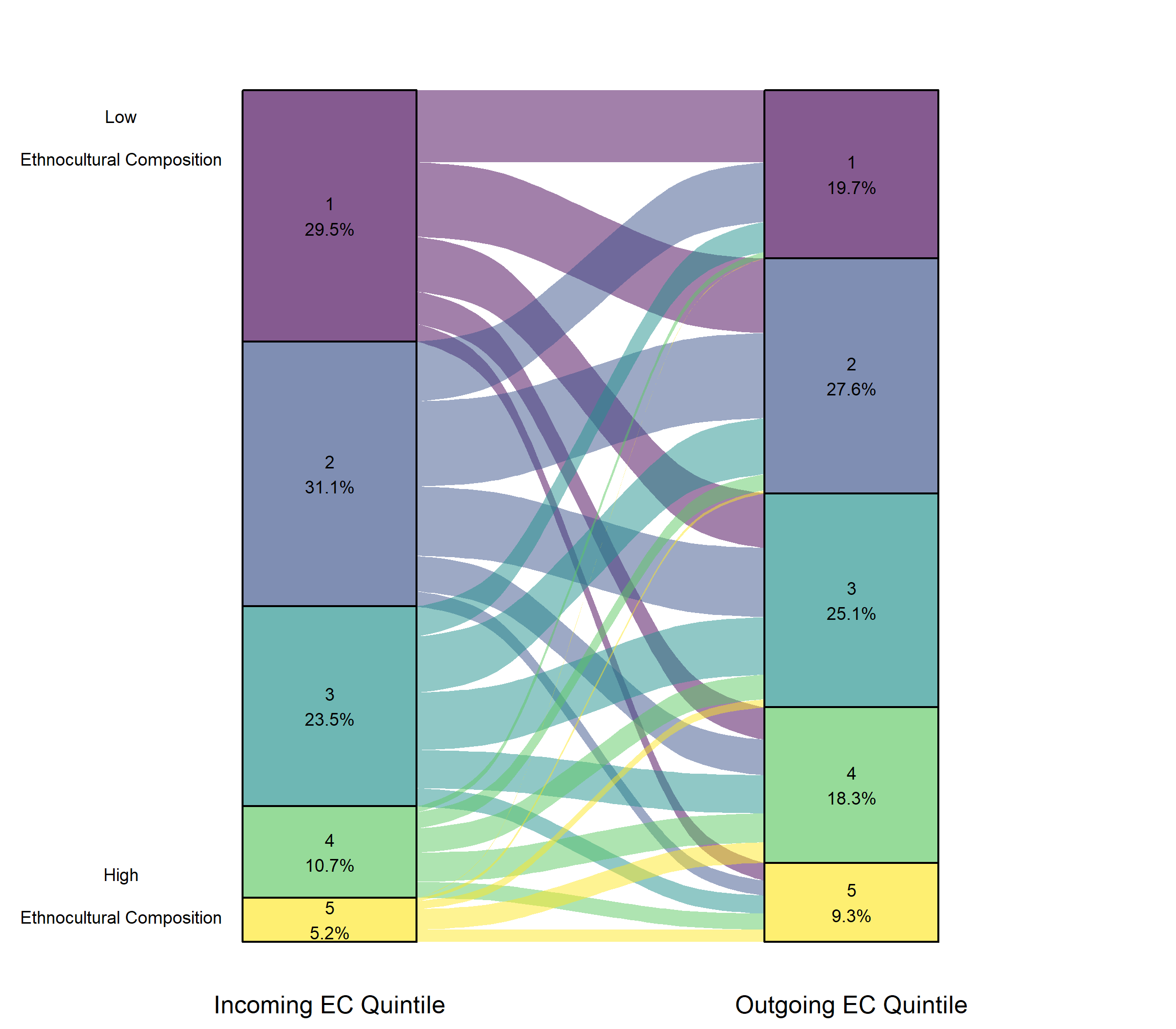


**Figure 2. Proportion of cats from each Situational Vulnerability quintile upon surrender (left axis) and upon adoption (right axis) for all intake groups adopted between January 1, 2016 to December 31, 2019 (n = 7,733).**
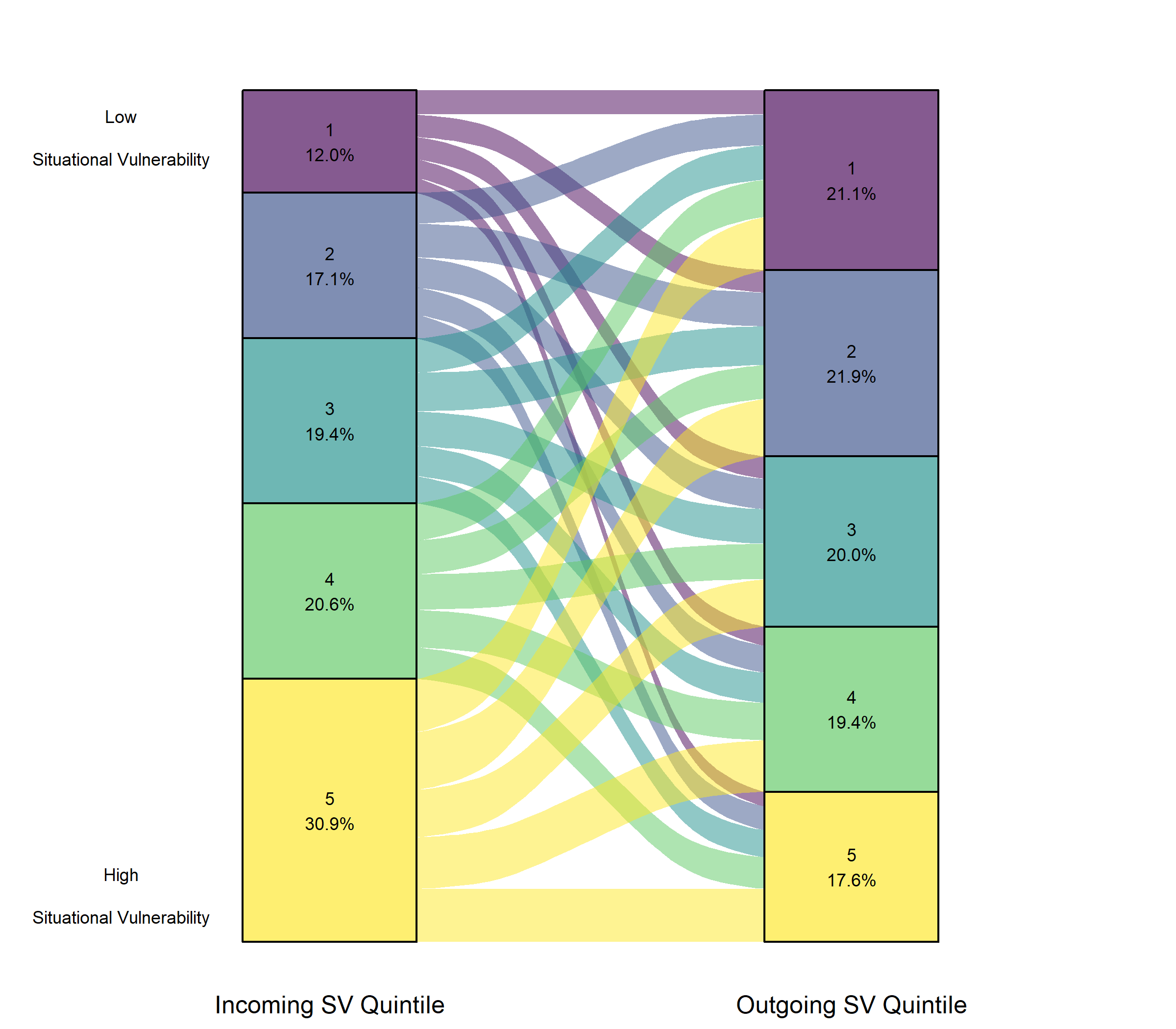


**Figure 3. Proportion of dogs from each Situational Vulnerability quintile upon surrender (left axis) and upon adoption (right axis) for all intake groups adopted between January 1, 2016 to December 31, 2019 (n = 3,446).**
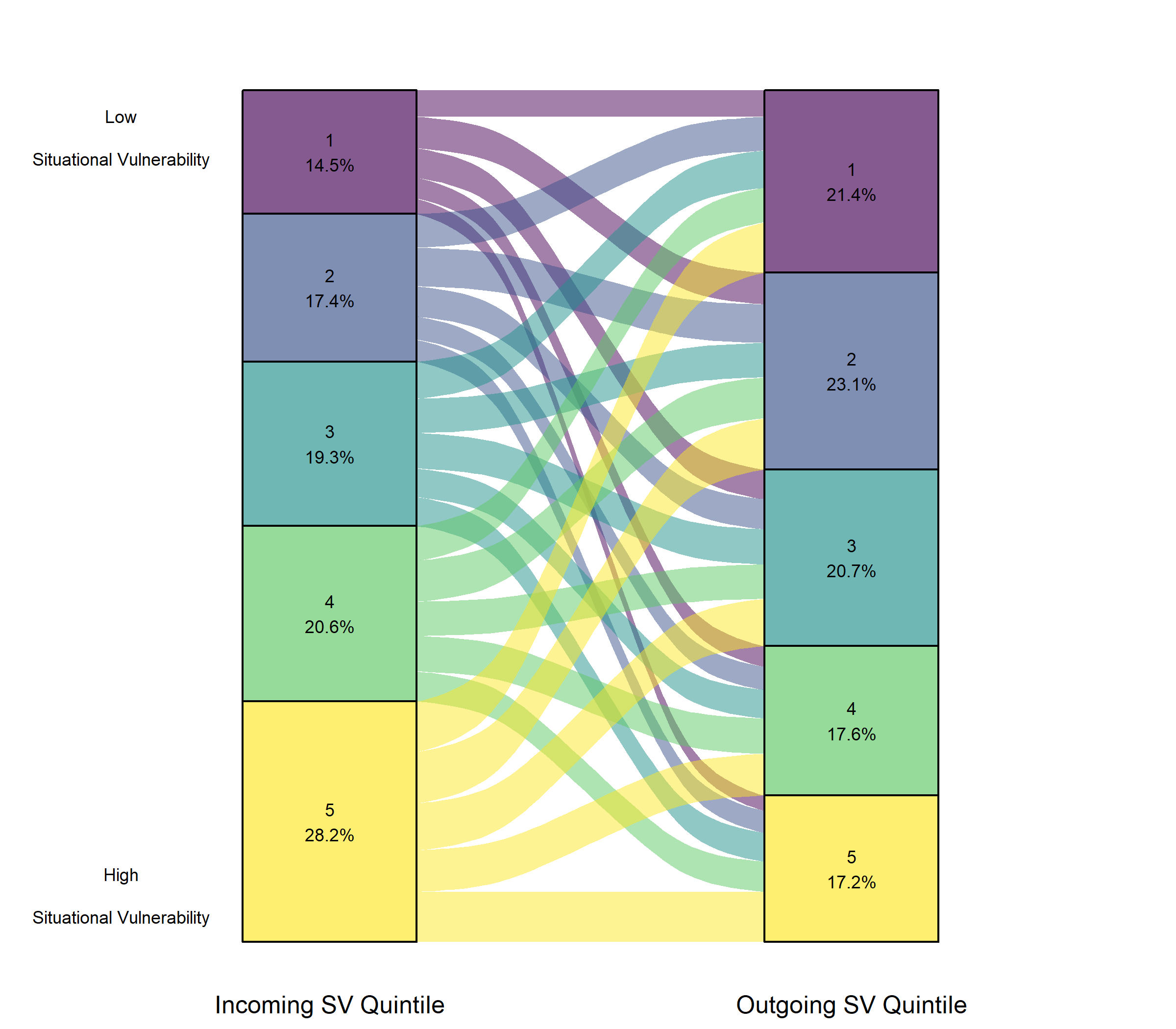


**Figure 4. Proportion of kittens from each Ethnocultural Composition quintile upon surrender (left axis) and upon adoption (right axis) for all intake groups adopted between January 1, 2016 to December 31, 2019 (n = 6,436).**
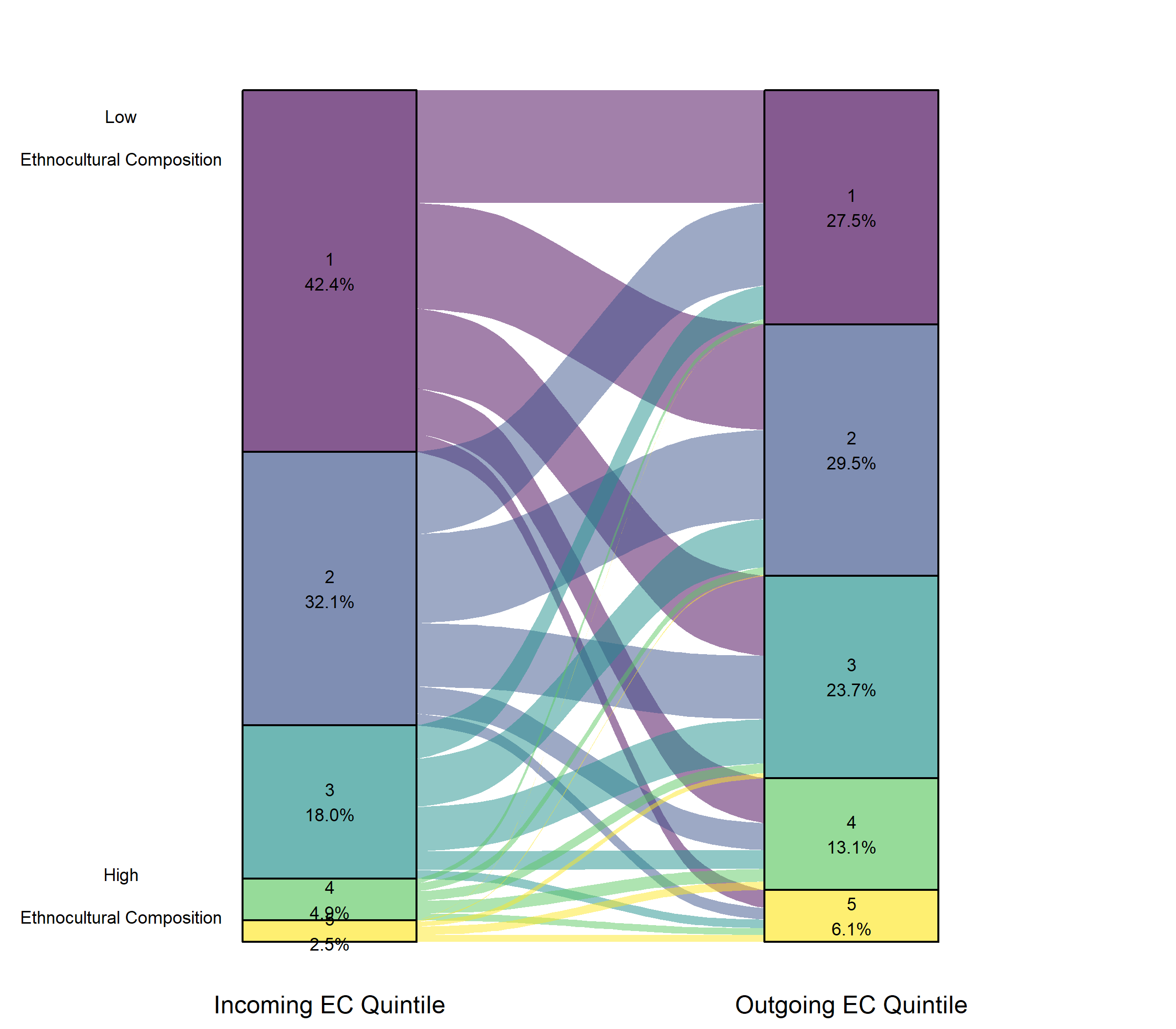


**Figure 5. Proportion of kittens from each Economic Dependency quintile upon surrender (left axis) and upon adoption (right axis) for all intake groups adopted between January 1, 2016 to December 31, 2019 (n = 6,436).**
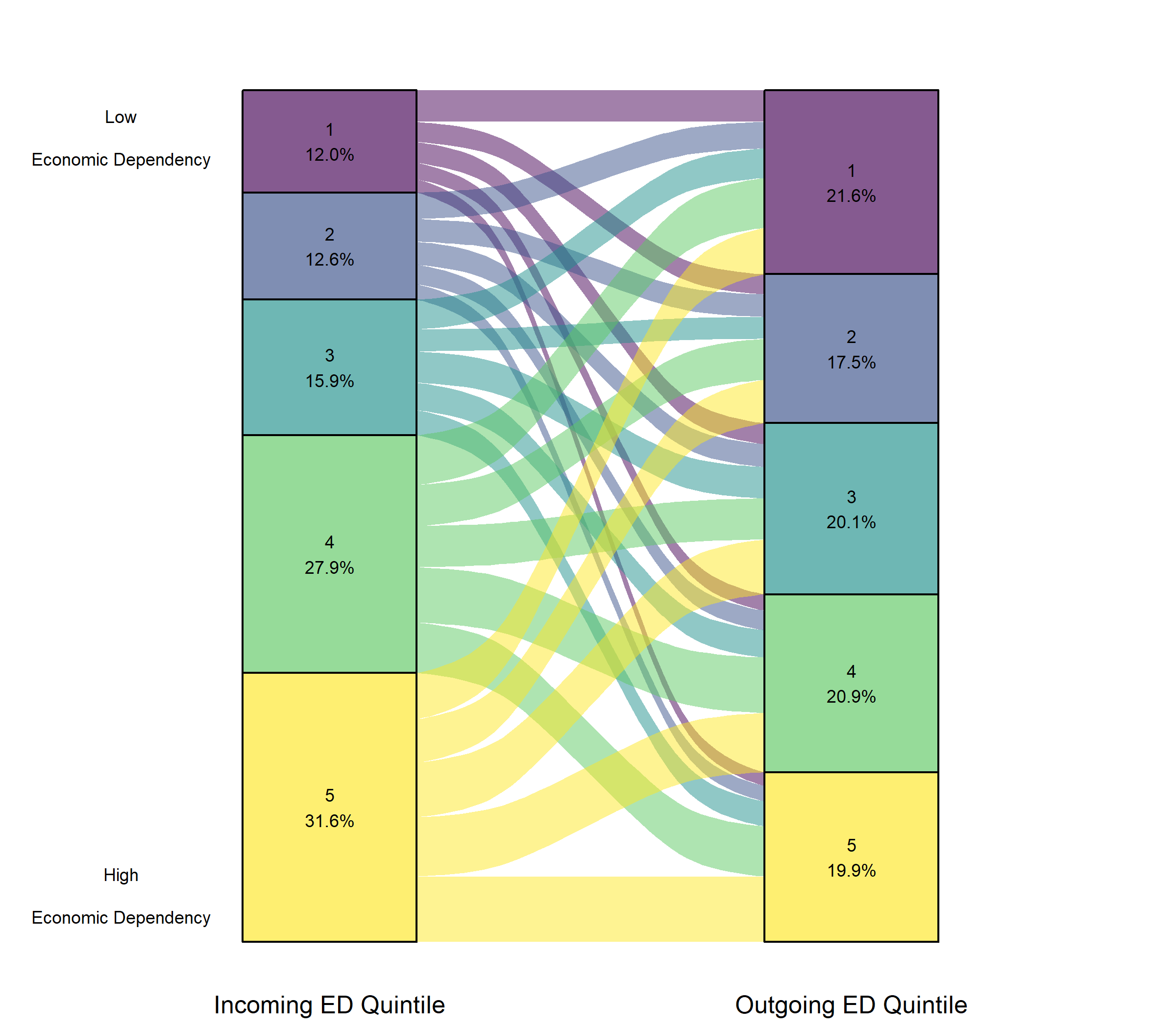


**Figure 6. Proportion of puppies from each Ethnocultural Composition quintile upon surrender (left axis) and upon adoption (right axis) for all intake groups adopted between January 1, 2016 to December 31, 2019 (n = 973).**
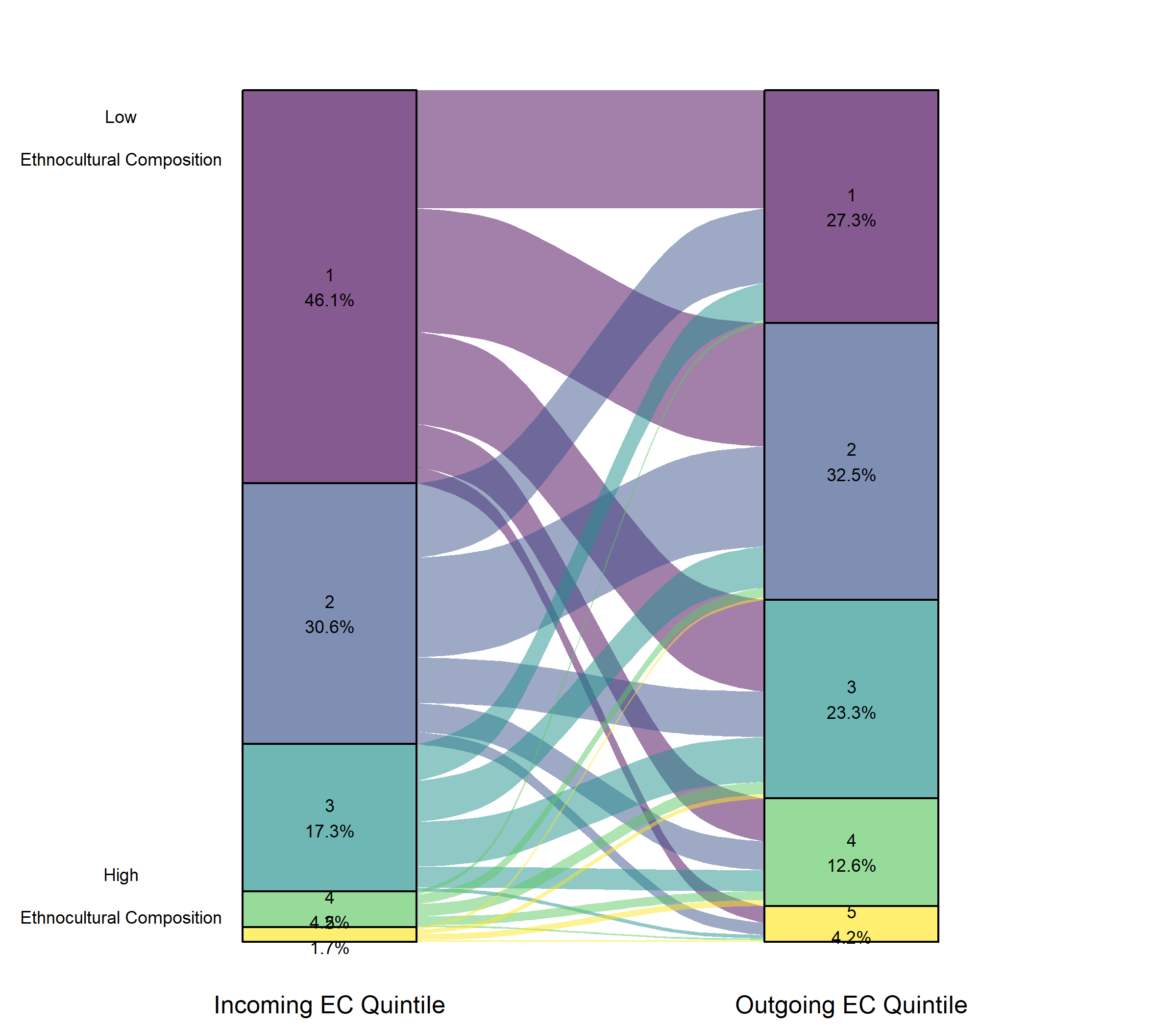


**Figure 7. Proportion of puppies from each Economic Dependency quintile upon surrender (left axis) and upon adoption (right axis) for all intake groups adopted between January 1, 2016 to December 31, 2019 (n = 973).**
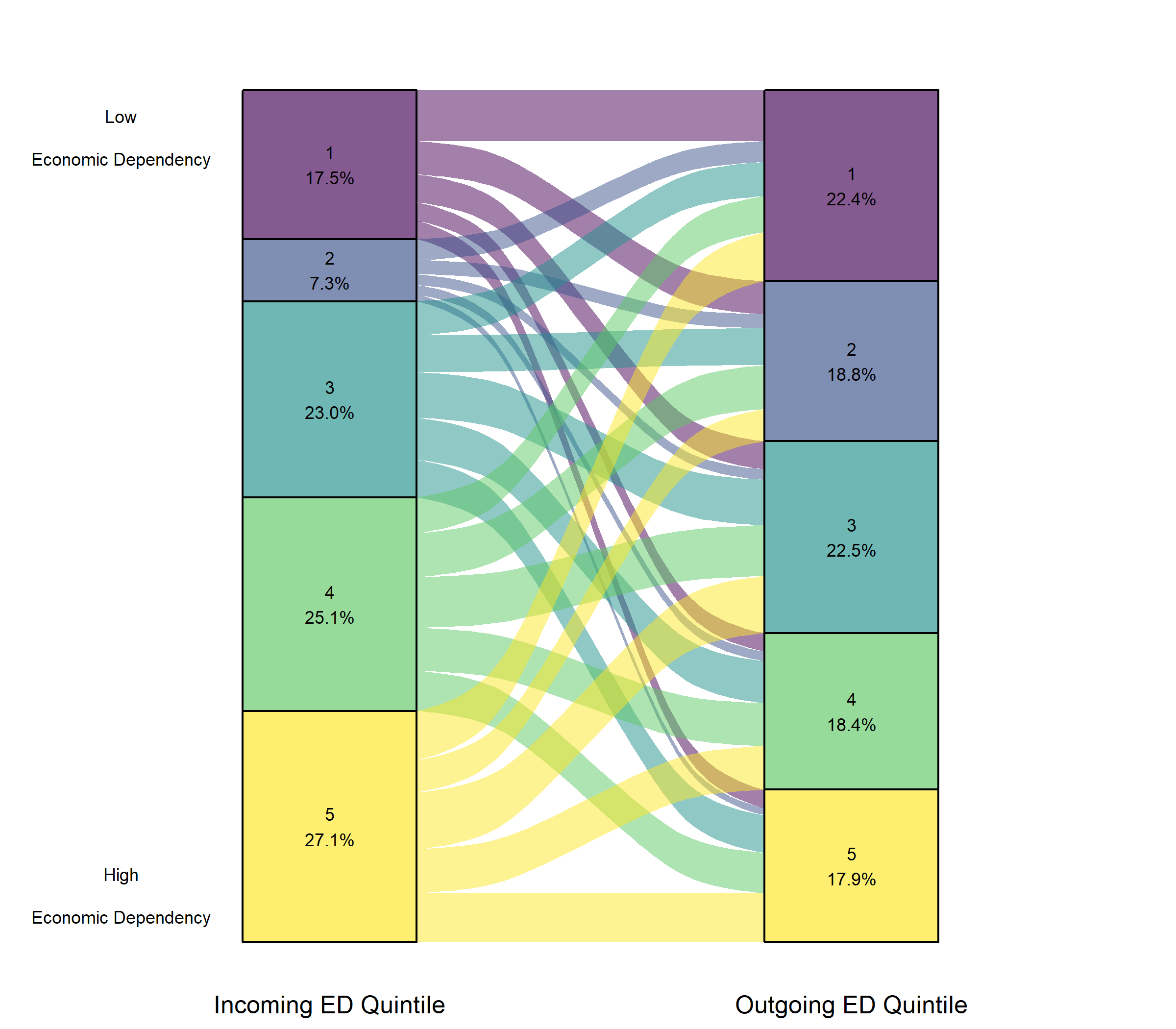


**Figure 8. Proportion of small animals from each Residential Instability quintile upon surrender (left axis) and upon adoption (right axis) for all intake groups adopted between January 1, 2016 to December 31, 2019 (n = 2,682).**


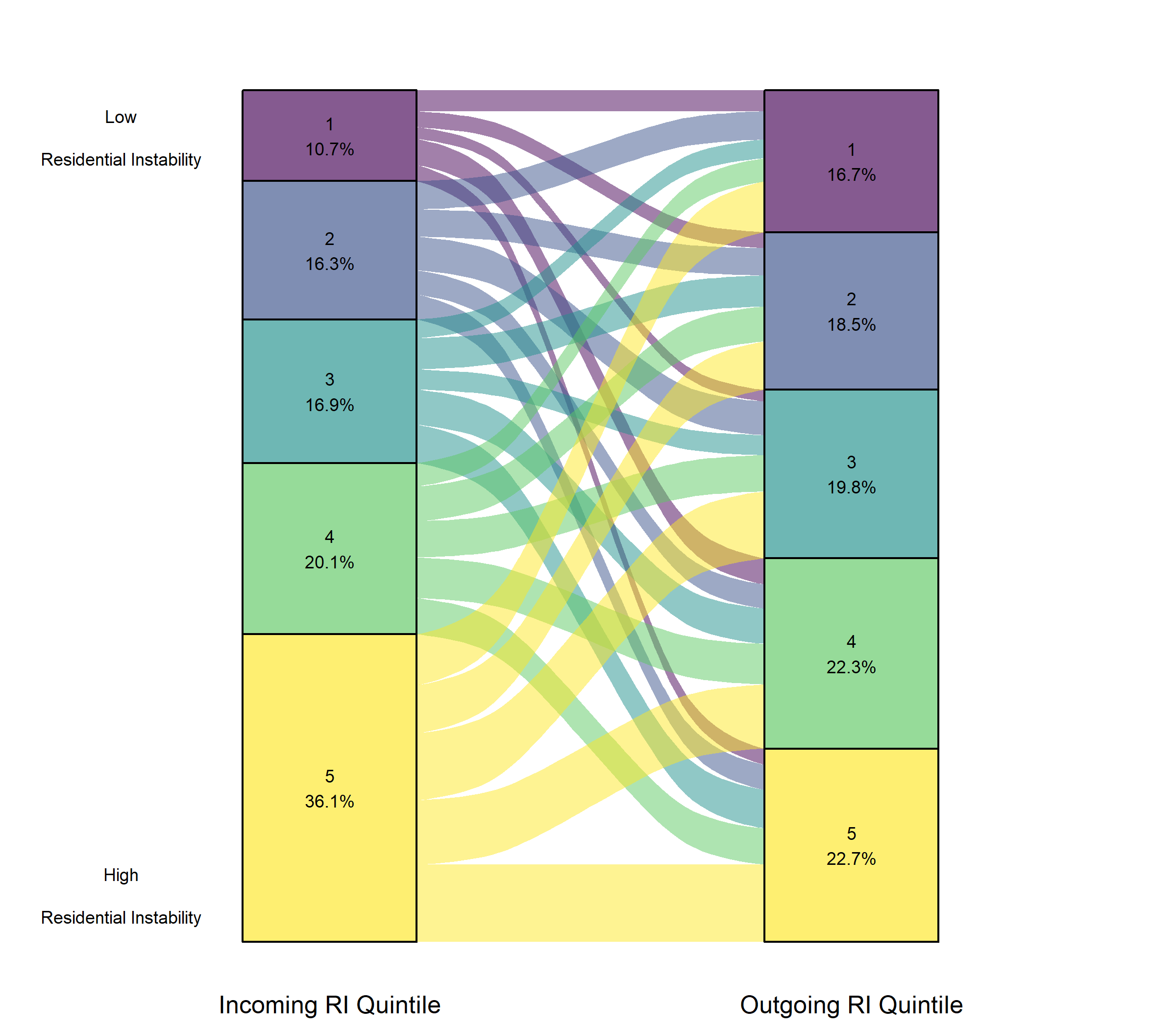

Supplement: Supplementary Data Sheet 2 — Supplementary alluvial plots showing the flow of animals with small effect size. [file Data_Sheet_2.docx]
